# Signal peptide mutations in RANK prevent downstream activation of NF-κB

**DOI:** 10.1002/jbmr.399

**Published:** 2011-04-06

**Authors:** Julie C Crockett, David J Mellis, Kathleen IJ Shennan, Angela Duthie, John Greenhorn, Debbie I Wilkinson, Stuart H Ralston, Miep H Helfrich, Michael J Rogers

**Affiliations:** 1 Musculoskeletal Research Program, University of Aberdeen Medical School, Institute of Medical SciencesForesterhill, Aberdeen, United Kingdom; 2 Cell and Developmental Research Program, University of Aberdeen Medical School, Institute of Medical SciencesForesterhill, Aberdeen, United Kingdom; 3 Rheumatic Diseases Unit, University of EdinburghEdinburgh, United Kingdom

**Keywords:** RANK, FAMILIAL EXPANSILE OSTEOLYSIS, EARLY-ONSET PAGET'S DISEASE, EXPANSILE SKELETAL HYPERPHOSPHATASIA, OSTEOCLAST, NFκB

## Abstract

Familial expansile osteolysis and related disorders are caused by heterozygous tandem duplication mutations in the signal peptide region of the gene encoding receptor activator of NF-κB (RANK), a receptor critical for osteoclast formation and function. Previous studies have shown that overexpression of these mutant proteins causes constitutive activation of NF-κB signaling in vitro, and it has been assumed that this accounts for the focal osteolytic lesions that are seen in vivo. We show here that constitutive activation of NF-κB occurred in HEK293 cells overexpressing wild-type or mutant RANK but not in stably transfected cell lines expressing low levels of each *RANK* gene. Importantly, only cells expressing wild-type RANK demonstrated ligand-dependent activation of NF-κB. When overexpressed, mutant RANK did not localize to the plasma membrane but localized to extensive areas of organized smooth endoplasmic reticulum, whereas, as expected, wild-type RANK was detected at the plasma membrane and in the Golgi apparatus. This intracellular accumulation of the mutant proteins is probably the result of lack of signal peptide cleavage because, using two in vitro translation systems, we demonstrate that the mutations in *RANK* prevent cleavage of the signal peptide. In conclusion, signal peptide mutations lead to accumulation of RANK in the endoplasmic reticulum and prevent direct activation by RANK ligand. These results strongly suggest that the increased osteoclast formation/activity caused by these mutations cannot be explained by studying the homozygous phenotype alone but requires further detailed investigation of the heterozygous expression of the mutant RANK proteins. © 2011 American Society for Bone and Mineral Research

## Introduction

Osteoclasts are the multinucleated giant cells responsible for bone resorption and are derived from circulating hematopoietic cells of the macrophage-monocyte lineage.^1^ Differentiation of these precursor cells into osteoclasts is critically dependent on the cell surface receptor activator of NF-κB (RANK), a member of the tumor necrosis factor receptor superfamily (encoded by *TNFRSF11A*).^2^, ^3^ Interaction of this 66-kDa integral membrane protein with RANK ligand (RANKL), expressed on stromal cells, osteoblasts, and activated T cells, causes trimerization of the receptor and initiates a signaling cascade that results in the activation of NF-κB and other transcription factors, leading to osteoclastogenesis.^4^–^7^

Mutations in RANK have been identified as the cause of familial expansile osteolysis (FEO), early-onset Paget disease of bone (ePDB), and expansile skeletal hyperphosphatasia (ESH), which are related disorders of bone metabolism characterized by focal areas of increased bone turnover together with a generalized increase in bone remodeling.^8^ Affected areas show an increase in osteoclast number, multinuclearity, and activity, which leads to the notion that these diseases are driven by osteoclast overactivity, as is classic late-onset Paget disease of bone.^9^ Heterozygous tandem duplication mutations in the signal peptide region of the *RANK* gene in patients with FEO (84dup18), ePDB (75dup27), or ESH (84dup15) result in extensions of 6, 9, or 5 amino acids, respectively, to the signal peptide region of the RANK protein.^10^, ^11^ The duplication in ePDB is only associated with early-onset familial Paget disease rather than the classic late-onset form of the disease.^12^ However, the exact molecular mechanism by which these alterations in the RANK signal peptide lead to the characteristic feature of osteoclast activation and enhanced bone turnover is unknown. Similar to other membrane proteins, overexpression of RANK in vitro causes constitutive activation of NF-κB.^13^, ^14^ Overexpression of FEO-RANK and PDB-RANK in EBNA-293 cells caused greater constitutive activation of NF-κB than wild-type RANK (WT-RANK) when normalized to the level of RANK protein.^10^ This suggested that ligand-independent activation of NF-κB could be a factor leading to the hyperactivated osteoclasts observed in vivo, although the mechanism underlying such activation was not examined. Therefore, the aim of this study was to clarify how the signal peptide tandem duplication mutations in RANK affect posttranslational processing and subcellular localization of RANK protein and the downstream activation of NF-κB.

## Materials and Methods

### Plasmids

pTREshuttle vector (BD Biosciences, Oxford, UK) has a minimal CMV promoter with a tetracycline response element. Transcription only occurs when the tetracycline-responsive transactivator expressed by the Tet-Off-HEK293 cell line binds the Tet-responsive element. pcDNA5-FRT vector has a CMV promoter and contains a Flp-recombinase transferase (FRT) site for integration of genes of interest into the genome of Flp-In-293 cells (Invitrogen, Paisley, UK). pOG44 encodes the Flp recombinase under the control of a CMV promoter. PDB-RANK (in pBluescript SKII) was a kind gift from Immunex Corporation (Amgen Inc, Thousand Oaks, CA, USA). All other materials were purchased from Sigma (Poole, UK) unless otherwise stated.

### Construction of pTREshuttle-RANK-FLAG vectors

A FLAG tag (GAC TAC AAG GAC GAC GAC GAC AAG) was inserted directly upstream of the PDBRANK stop codon by site-directed mutagenesis (SDM; Quik-Change SDM Kit; Stratagene, Amsterdam, The Netherlands). WT-RANK-FLAG, FEO-RANK-FLAG, and ESH-RANK-FLAG then were produced by polymerase chain reaction (PCR)–based cloning from genomic wild-type human DNA into the SalI/PstI sites (WT-RANK-FLAG) or by SDM (FEO-RANK-FLAG and ESH-RANK-FLAG). These then were ligated into the ApaI/NotI sites of the pTREshuttle vector (BD Biosciences) using T4 DNA ligase (Roche Diagnostics, Ltd., Lewes, UK).

### Construction of recombinant adenoviruses

Recombinant adenoviruses containing WT-RANK-FLAG, FEO-RANK-FLAG, and PDB-RANK-FLAG were generated by ligation using the Adeno-X-TetOff expression system (Takara Clontech, Mountain View, CA, USA) following the manufacturer's instructions. Briefly, the *RANK* cDNAs were excised from pTREshuttle using PI-SceI and I-Ceu-I restriction enzymes and ligated into linearized adenoviral DNA. The adenoviral DNA, containing the *RANK* cDNA, was purified using a large-construct DNA purification kit (Qiagen, Crawley, UK). Following PacI digestion to linearize the adenoviral DNA, recombinant adenoviruses then were produced by transfecting DNA into HEK293 cells by calcium phosphate precipitation. After 4 to 7 days, the adenovirus was harvested from the cells by rapidly freeze-thawing the cells. Primary amplification adenoviral stocks were prepared by amplification in HEK 293 cells cultured in 175-cm^2^ tissue culture flasks, and these stocks were titered by endpoint dilution assay. Typical titers were greater than 1 × 10^9^ pfu/mL.

### Construction of pcDNA-RANK-FLAG-FRT vectors and generation of stable cell lines

The WT-RANK-FLAG, FEO-RANK-FLAG, PDB-RANK-FLAG, and ESH-RANK-FLAG were ligated into the *Bam*H I/Not I sites in pcDNA5-FRT vector using T4 DNA ligase. The Flp-In system (Invitrogen) was used to generate cell lines (Flp-WT, Flp-FEO, Flp-PDB, and Flp-ESH) incorporating a single copy of each of the RANK-FLAG cDNAs. Flp-In-293 cells (that stably express a FRT site) were cultured in Dulbecco's modified Eagle medium (DMEM) containing 4500 mg/mL of glucose and supplemented with 10% (v/v) fetal calf serum (FCS), 2 mM L-glutamine, 100 U/mL of penicillin, 100 µg/mL of streptomycin, 100 µg/mL of zeocin. Flp-In-293 cells in 10-cm^2^ tissue culture plates were transfected with 1 µg of pcDNA5-RANKFLAG plasmid together with 9 µg of pOG44 plasmid by Fugene 6 (Roche Diagnostics, Ltd.) transfection. Stably transfected cells were selected in medium containing 100 µg/ml of hygromycin, and clones were selected that were hygromycin-resistant, zeocin-sensitive, and *LacZ*^−^. Genomic DNA and RNA were isolated from each clone, and the presence and expression of *RANK-FLAG* genes were confirmed by Big Dye sequencing from PCR products amplified using primers across exons 1 to 3 to amplify the region containing the duplication mutations (fwd: 5'-gga tcc aga cat gat aag gg-3', which was anchored within the region of the pcDNA5FRT expression vector immediately upstream of the transcription start to ensure amplification of the exogenous *RANK* genes; and rev: 5'-atc caa gta ttc atc cgg gcc c-3', within exon 3 of the *RANK* gene), and primers that specifically amplified the *FLAG* region of the inserted *RANK* genes (fwd: 5'-aga aga agc cag cag gac gga-3'; and rev: 5'-gct cac ttg tcg tcg tcg tcc-3', anchored within the *FLAG* tag). Expression of endogenous RANK within the parental Flp-In-293 cell line was confirmed by RT-PCR using one set of primers to amplify a region across exons 5 to 7 of *RANK* (fwd: 5'-tgc agc tca aca agg aca cag-3'; and rev: 5'-gct gtg agt gct ttc cct ttt-3') and another set to amplify a region within exon 9 (fwd: 5'-ttc acg ggg aca cag agc aca-3'; and rev: 5'-cct ccg tcc tgc tgg ctt cttc-3'). Finally, to compare the relative level of expression of *RANK* in the stable cell lines compared with 293 cells that had been transfected with the pcDNA5FRT-RANK expression constructs, quantitative RT-PCR was performed on a LightCycler 480 (Roche Applied Science, Basel, Switzerland) using Universal Probes Mastermix (Applied Biosystems, Foster City, CA, USA), gene-specific primers (fwd: 5'-gaa cat cat ggg aca gag aaa-3'; and rev: 5'-ggc aag taa aca tgg ggt tc-3') and Universal Probe Number 53 (Applied Biosystems). *GAPDH* was used as the housekeeping gene and was amplified simultaneously in parallel samples using the *GAPDH* assay mix (human) (Applied Biosystems). *RANK* expression was normalized to *GAPDH,* and expression relative to that in untransfected cells was calculated.

### Cell culture and transfections

Tet-Off 293 cells (BD Biosciences) were cultured, as advised by the supplier, on collagen-I-coated tissue culture vessels (Sigma, Poole, UK) in α-modified minimum essential medium (α-MEM) containing 1 mM glutamine, 100 U/mL of penicillin, 100 µg/mL of streptomycin, 10 µg/mL og G418, and 10% (v/v) Tet-approved fetal bovine serum (FBS; BD Biosciences). 293 cells [European Cell and Culture Collection (ECACC)] were cultured in α-MEM containing 1 mM glutamine, 100 U/mL of penicillin, 100 µg/mL of streptomycin, and 10% (v/v) FCS.

Transfections were carried out using Fugene-6 (Roche Diagnostics, Ltd.) approximately 18 hours after seeding. For Western blot analysis, 293 cells were seeded into 12-well plates at 4 × 10^5^ cells/well, transfected with 0.25, 0.5, and 1 µg of pcDNA5-RANK-FLAG plasmids or pcDNA5FRT (empty vector) and cultured for 24 hours. For immunostaining, 293 cells were cultured on glass coverslips in 24-well plates at 1 × 10^5^ cells per well, transfected with 0.5 µg of pcDNA5-RANK-FLAG or pcDNA5FRT (empty vector), and cultured for 48 hours. For reporter assays to assess the effect of overexpression of the RANK constructs on NF-κB activation, Tet-Off 293 cells or Flp-RANK cell lines were seeded into 48-well plates at 1 × 10^5^ cells/well and transfected as described for each experiment.

### Generation of human osteoclast-like cells and transduction with *RANK* recombinant adenoviruses

Ethical permission for the culture of cells derived from the blood of healthy volunteers was obtained from the North of Scotland Research Ethics Committee. Peripheral blood mononuclear cells were isolated from human peripheral blood by Lymphoprep density centrifugation and cultured for 7 days in the presence of 20 ng/mL of recombinant human macrophage colony-stimulating factor (rhM-CSF; R&D Systems, Minneapolis, MN, USA) to give cultures of enriched M-CSF-dependent monocytes. Cells were removed from the flasks by trypsinization for 30 minutes in trypsin/EDTA and scraped gently into α-MEM supplemented with 10% (v/v) FCS, 1 mM L-glutamine, 100 U/mL of penicillin, 100 µg/mL of streptomycin, and 20 ng/mL of rhM-CSF. They then were seeded onto 9-mm coverslips (preincubated in FCS for 30 minutes) in 48-well plates at a density of 3 × 10^4^ cells/well or onto Whatman 0.45-µm nitrocellulose filter paper in 96-well plates and cultured in 100 ng/mL of rhRANKL (Pepro-Tech, Rocky Hill, NJ, USA) and 20 ng/mL of rhM-CSF for about 5 days until the appearance of large, multinucleated osteoclast-like cells in the 48-well plates. The medium was removed, and the cells were incubated for 3 hours in 200 pfu/cell Tet regulatory adenovirus together with 200 pfu/cell WT-RANK-FLAG, FEO-RANK-FLAG, or PDB-RANK-FLAG adenoviruses in 100 µL (48-well plates) or 50 µL (96-well plates) serum-free α-MEM supplemented with 1 mM L-glutamine, 100 U/mL of penicillin, 100 µg/mL of streptomycin, 20 ng/mL of rhM-CSF, and 100 ng/mL of rhRANKL. The plates were incubated for 3 hours and then supplemented with 100 µL (48-well plates) or 50 µL (96-well plates) of α-MEM supplemented with 20% Tet-approved FCS, 1 mM L-glutamine, 100 U/mL of penicillin, 100 µg/mL of streptomycin, 20 ng/mL of rhM-CSF, and 100 ng/mL of rhRANKL. The cells were incubated for 48 hours and then fixed for immunostaining and confocal microscopy or for TEM analysis.

### Detection of WT-, FEO-, PDB-, or ESH- RANK-FLAG expression by Western blot analysis

Twenty-four hours after transfection, cells were prepared for Western blot analysis. The cells were washed in ice-cold PBS and lysed in 100 µL of radioimmunoprecipitation assay (RIPA) buffer [1% (vol/vol) NP-40, 0.5% (wt/vol) sodium deoxycholate, 0.1% (wt/vol) SDS in PBS) containing 1% protease inhibitor cocktail (Sigma). The lysates were vortexed and incubated on ice for 15 minutes. Insoluble material was removed by centrifugation (13,000 × *g*, 15 minutes). After protein determination (Bicinchoninic Acid Assay; Sigma), 50 µg of protein from each lysate were electrophoresed under reducing conditions on a 12% polyacrylamide-SDS resolving gel. The proteins were blotted onto polyvinylidenedifluoride membrane, which was incubated with 0.5 µg/mL of mouse monoclonal anti-RANK antibody (IMG-128; Imgenex Corporation, San Diego, CA, USA), 5 µg/mL of rabbit anti-FLAG antibody (Sigma) followed by infrared-labeled anti-mouse-800 (Licor Biosciences, Cambridge, UK) and anti-rabbit-680 (Invitrogen) secondary antibodies. The infrared signals were detected using the Odyssey imaging system (Licor Biosciences).

### Detection of WT-, FEO-, PDB-, or ESH-RANK-FLAG expression by immunostaining

Twenty-four or 48 hours after transfection/transduction, the cells were fixed for 20 minutes in CytoFix-CytoPerm (BD Biosciences) at 4°C and then washed in CytoFix-CytoPerm wash solution, blocked in 10% (vol/vol) FBS (in PBS), and incubated in 20 µg/mL of mouse monoclonal anti-FLAG antibody (Sigma), followed by 20 µg/mL of Alexafluor antimouse-IgG 594 (Molecular Probes, Leiden, The Netherlands). Cells were also stained with a Golgi marker (10 µg/mL of wheatgerm aggluttinin-633 (WGA-633); Invitrogen), the nuclei were counterstained with 10 µM Sytox Green (Invitrogen), and the coverslips were mounted in Vectashield (Vector Laboratories, Peterborough, UK). The cells were visualized on an LSM510 Meta confocal microscope (Carl Zeiss, Ltd., Welwyn Garden City, UK), and images were captured using LSM image software (Carl Zeiss, Ltd.).

### Detection of *RANK-FLAG* expression by immunoEM

293 cells in 6-well plates were transiently transfected with 1 µg WT-, FEO-, or PDB-RANK-FLAG using Fugene-6. The cells were incubated for 24 hours and then were fixed, embedded, and sectioned for transmission electron microscopy (TEM). The cells were pelleted by centrifugation and then fixed for 1 hour in 4% paraformaldehyde + 0.1% glutaraldehyde in 0.1 M sodium cacodylate buffer. Cell pellets then were cut in two. One-half was placed into 2% glutaraldehyde in 0.1 M sodium cacodylate buffer for processing to Epon resin for conventional TEM. The other half was placed in fresh 4% paraformaldehyde and processed to Lowicryl HM20 resin using a progressive lowering-of-temperature protocol in a Leica AFS2. RANK-FLAG proteins were then detected on ultrathin sections using 4 µg/mL of rabbit anti-FLAG antibody followed by 1:20 dilution of 10 nm of Protein A Gold (Aurion, Wageningen, The Netherlands). All sections (Epon and Lowicryl) were counterstained with uranylacetate and lead citrate and examined in a Philips CM10 microscope and images captured using a 600-W camera (Gatan, Abingdon, UK).

### Reporter assays

To determine the effect of the mutations on constitutive NF-κB activation, Tet-Off 293 cells were transfected with 1, 5, 10, and 30 ng of pTRERANK-FLAG plasmids or 30 ng of pTREshuttle together with 60 ng of pNF-κB reporter plasmid (8 replicate wells per transfection). To determine the effect of the mutations on constitutive and RANKL-dependent NF-κB activation, Tet-Off 293 cells were transfected with 5 ng of pTRERANK-FLAG plasmids or 5 ng of pTREshuttle together with 60 ng of pNF-κB reporter plasmid (Takara Clontech) and 10 ng of pshuttle *LacZ* (8 replicate wells per transfection). After 24 hours, 4 wells of each transfected cell culture were stimulated with 100 ng/mL of RANKL (Pepro-Tech) for a further 24 hours. To determine the effect of the mutations on RANKL-dependent activation of NF-κB in cells that stably express low levels of the RANK proteins, Flp-RANK cells were transfected with 150 ng of pNF-κB reporter plasmid and 150 ng of pTKRenilla (8 replicate wells per transfection). After 24 hours, 4 wells of each transfected cell culture were stimulated with 100 ng/mL of RANKL (PeproTech) for a further 24 hours.

In all cases, after treatment, the cells were washed in PBS and lysed in 40 µL of passive lysis buffer (Promega UK, Ltd., Southampton, UK). The samples were analyzed for firefly and either *Renilla* luciferase activity using the Dual Luciferase Assay Kit (Promega) and the Wallac 1420 Victor^2^ multilabel plate reader (Perkin Elmer, Cambridge, UK), or β-galactosidase expression was measured using the β-galactosidase assay (Promega) by absorbance at 405 nm on a Bio-Tek FL600 plate reader (Bio-Tek Instruments, Inc., Winooski, VT, USA).

### In vitro translation assays

WT-, FEO-, PDB-, and ESH-RANK-FLAG were cloned into the *ApaI*/*NotI* sites in a modified SP64T vector (Krieg and Melton, 1984) that allows transcription of mRNA that is flanked by the 5' and 3' untranslated regions of *β-globin* RNA to improve stability of the mRNA in the translation systems. The RANK-SP64T vectors (20 µg) were linearized with *Xho1*, and cRNA was prepared as described previously.^15^ To determine whether signal peptide cleavage occurs in each protein, the mass of proteins that had been translated in the XEE (in the presence of NYT) was compared with that translated in the RRL. The XEE translation was carried out as previously described^15^ using 1 µL of H_2_O or RNA encoding either WT-, FEO-, PDB-, or ESH-RANK-FLAG in the presence or absence of 1.2 mM tripeptide (acetyl)-Asn-Tyr-Thr-(amide) (NYT) to inhibit glycosylation. RRL translations were carried out following manufacturer's instructions (Promega) using 1 µL of H_2_O or WT-, FEO-, PDB-, or ESH-RANK RNA and allowed to proceed for 1 hour. All translations were analyzed on an 8% SDS-polyacrylamide (Tris-glycine buffer) gel. Following fluorography, the gel was exposed to film overnight at −80°C.

**Fig. 7:**
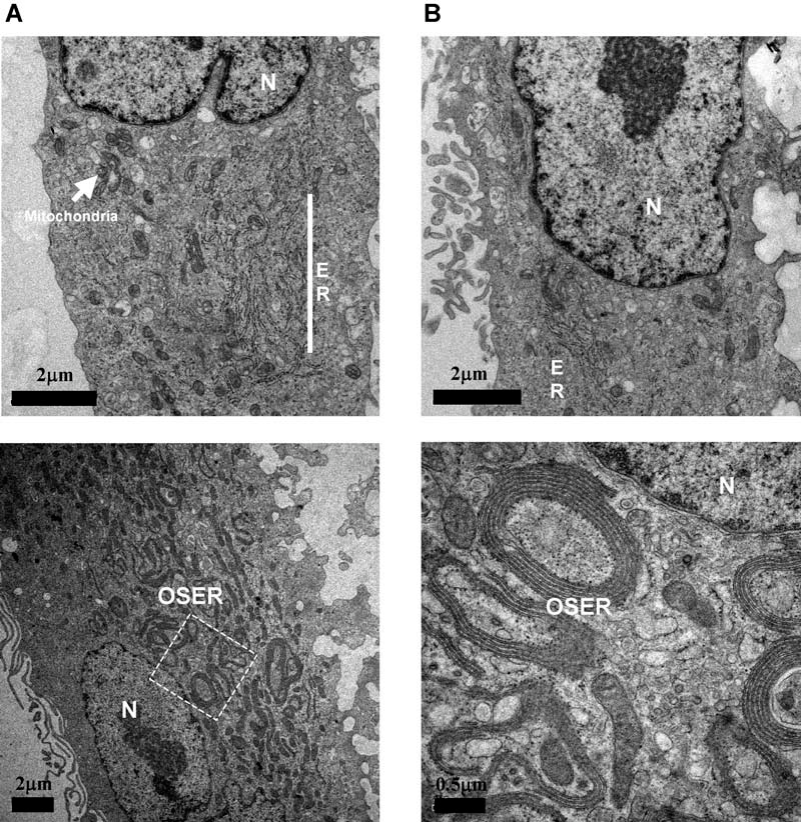
TEM analysis demonstrates that overexpression of FEO-RANK-FLAG in human osteoclasts results in OSER formation. Human osteoclasts that had been transduced with (*A*) adeno-WT-RANK-FLAG show normal cellular ultrastructure. When cells were transduced with (*B*) adeno-FEO-RANK-FLAG, OSER formation can be seen clearly. Scale bars are shown on each image. N = nucleus; ER = endoplasmic reticulum; OSER = organized smooth endoplasmic reticulum.

To determine the orientation of each protein in the in vitro membranes, a protease protection assay was carried out (in a XEE translation), as described previously,^15^ using 1 µL of RNA encoding either WT-, FEO-, PDB-, or ESH-RANK-FLAG. Within an XEE translation, proteins are inserted into the membrane vesicles so that the protein domains that would be expected to be intracellular are on the outside face of the vesicles, and the extracellular domain of the protein is within the vesicle (as represented in the schematic diagram in Fig. 7*B*). This means that when the vesicles that carry the proteins are exposed to proteinase K, the intracellular domain of the protein will be degraded, and the extracellular domain will be protected and still will be intact when the products are analyzed by PAGE and fluorography. Proteinase K activity was inhibited by addition of PMSF before the samples were analyzed on a 12.5% SDS-polyacrylamide (Tris-glycine buffer) gel. Following fluorography, the gel was exposed to film overnight at −80°C.

### Statistical analysis

Statistical significance was calculated using a one-way ANOVA with Tukey post hoc test.

## Results

### FEO-RANK, PDB-RANK, and ESH-RANK proteins are of higher molecular mass and are expressed at lower levels than WT-RANK

**Fig. 1:**
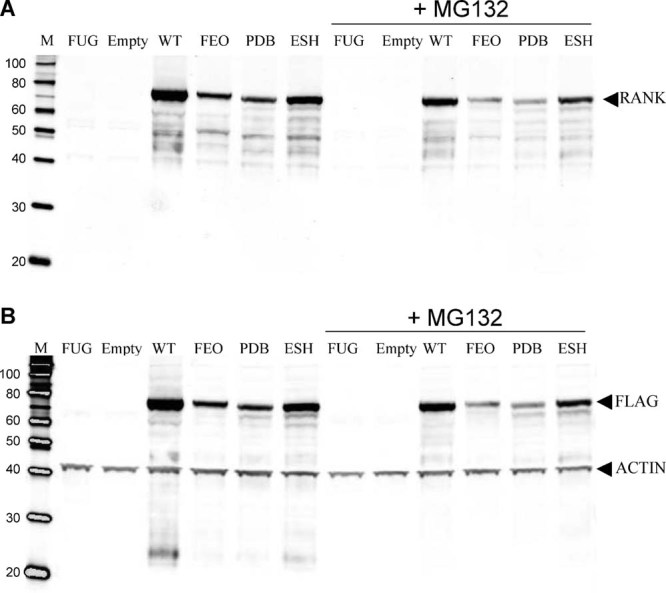
WT-RANK-FLAG is of lower molecular mass and is more abundant than mutant RANK-FLAG protein, which is not as a result of increased proteasomal degradation of the mutant proteins. Infrared (Licor) detection of (*A*) RANK (800 nm) and (*B*) FLAG and actin expression (680 nm) on the same Western blot of 293 cells that had been mock transfected 48 hours previously (CTL) or transfected with 0.5 µg of pcDNA5FRT (EV), WT-RANK-FLAG, FEO-RANK-FLAG, PDB-RANK-FLAG, or ESH-RANK-FLAG in the presence or absence of 1 µM MG132 for the final 24 hours of culture. Data are representative of three independent experiments.

To confirm expression of WT-, FEO-, PDB-, and ESH-RANK, HEK 293 cells were transfected with *RANK-FLAG* expression plasmids, and the RANK proteins were detected 48 hours later by Western blot analysis using anti-RANK monoclonal (mouse) and anti-FLAG polyclonal (rabbit) antibodies. WT-RANK was identified as a band of approximately 70 kDa (Fig. 1), whereas the bands corresponding to FEO-, PDB-, and ESH-RANK were slightly greater. The level of FEO-RANK, ESH-RANK, and PDB-RANK protein in lysates of transiently transfected cells was lower than that of WTRANK when normalized to the level of β-actin. To determine whether the apparently lower levels of mutant RANK versus WTRANK were as a result of increased proteasomal degradation, cells were treated with 1 µM MG132 for the final 24 hours of culture. This did not increase the relative levels of any of the proteins.

### Expression levels of RANK in Flp-WT, Flp-FEO, Flp-PDB, and Flp-ESH cell lines are much lower than in cells that have been transiently transfected with *RANK* constructs

**Fig. 2:**
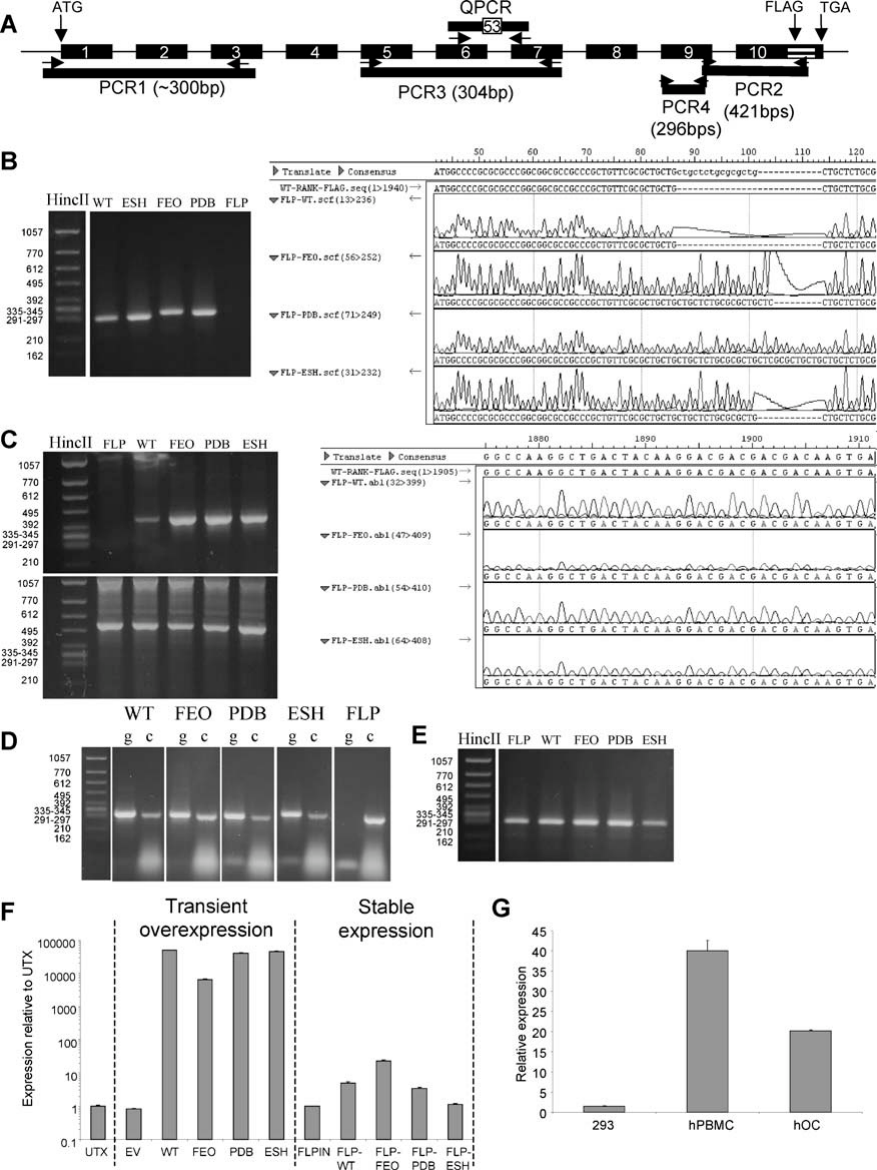
Confirmation of RANK expression in Flp-In cell lines by RTPCR, qPCR, and sequencing using construct-specific primers (*A*). The region around the tandem duplications in *RANK* was amplified by PCR 1 from genomic DNA from all Flp-In cell lines (*B*, *left panel*), and the products were sequenced and aligned to a consensus WT-RANK sequence to confirm that each cell line contained the expected duplication (*B*, *right panel*: FEO 18 bp, PDB 27 bp, ESH 15 bp). The region around the *FLAG* tag was amplified by PCR 2 from genomic DNA from all Flp-In cell lines (*C*, *left panel*, *top*) with primers to amplify *β-actin* as housekeeping gene (*C*, *left panel*, *bottom*). The products were sequenced and aligned to a consensus RANK-FLAG sequence to confirm the presence of the C-terminal end of the RANK-FLAG constructs within each cell line (*C*, *right panel*). (*D*) PCR 3 was performed on genomic (g) and cDNA synthesized from RNA (c) extracted from all Flp-In cell lines, and PCR 4 was performed on genomic DNA (*E*). All PCR products were analyzed by ethidium bromide agarose gel electrophoresis with *Hin*cII DNA size markers, and the expected band sizes for each PCR reaction are shown in *A*. Data from QPCR (UPL probe 53) for (*F*) 293 cells that had been transiently transfected with RANK constructs or Flp-In cell lines stably expressing the *RANK-FLAG* genes and (*G*) 293, human peripheral blood mononuclear cells, and human osteoclast-like cells. The data represent the ΔΔ*CP* values normalized to *GAPDH* relative to expression levels in untransfected (UTX) cells. All qPCR data are representative of two independent experiments (three replicates per experiment). Note the log scale on the *y* axis in *F*.

Integration of the wild-type and mutant *RANK-FLAG* constructs into the FRT site within the Flp-In-293 cell line was confirmed by hygromycin resistance, zeocin sensitivity, and loss of β-galactosidase expression (data not shown). Sequencing data of regions within exons 1 to 3 and across the *FLAG* tag (Fig. 2*A*) confirm that within each cell line the relevant tandem duplication mutation and *FLAG* tag is present (Fig. 2*B*, *C*). Using a pair of primers that amplified exons 3 to 7, *RANK* could be detected in the genomic DNA from Flp-WT, Flp-FEO, Flp-PDB, and Flp-ESH but not in the parental Flp-In cell line, whereas within the cDNA synthesized from the same cell lines, *RANK* was detected in all cases, as expected (Fig. 2*D*). In all cell lines, including Flp-In-293 cells, a product was detected using primers that amplified a region within exon 9 (Fig. 2*E*). Quantitative polymerase chain reaction (qPCR) using *RANK*-specific primers together with a UPL probe confirmed that in transient overexpression experiments, when normalized to *GAPDH*, levels of *RANK* expression were between 6500 and 50,000 times greater than levels of *RANK* expression in the untransfected cells, whereas in the stable cell lines, relative *RANK* expression levels were between 1.1 and 23 times greater than in the parental Flp-In-293 cell line (Fig. 2*F*). In addition, the expression levels of *RANK* in 293 cells was very low compared with human peripheral blood mononuclear cells and human osteoclast-like cells, in which expression levels were approximately 40 and 20 times more than in 293 cells, respectively (Fig. 2*G*).

### Overexpression of WT-RANK-FLAG, FEO-RANK-FLAG, PDB-RANK-FLAG, and ESH-RANK-FLAG causes constitutive activation of NF-κB, but only cells overexpressing WTRANK show RANKL-dependent activation of NF-κB

**Fig. 3:**
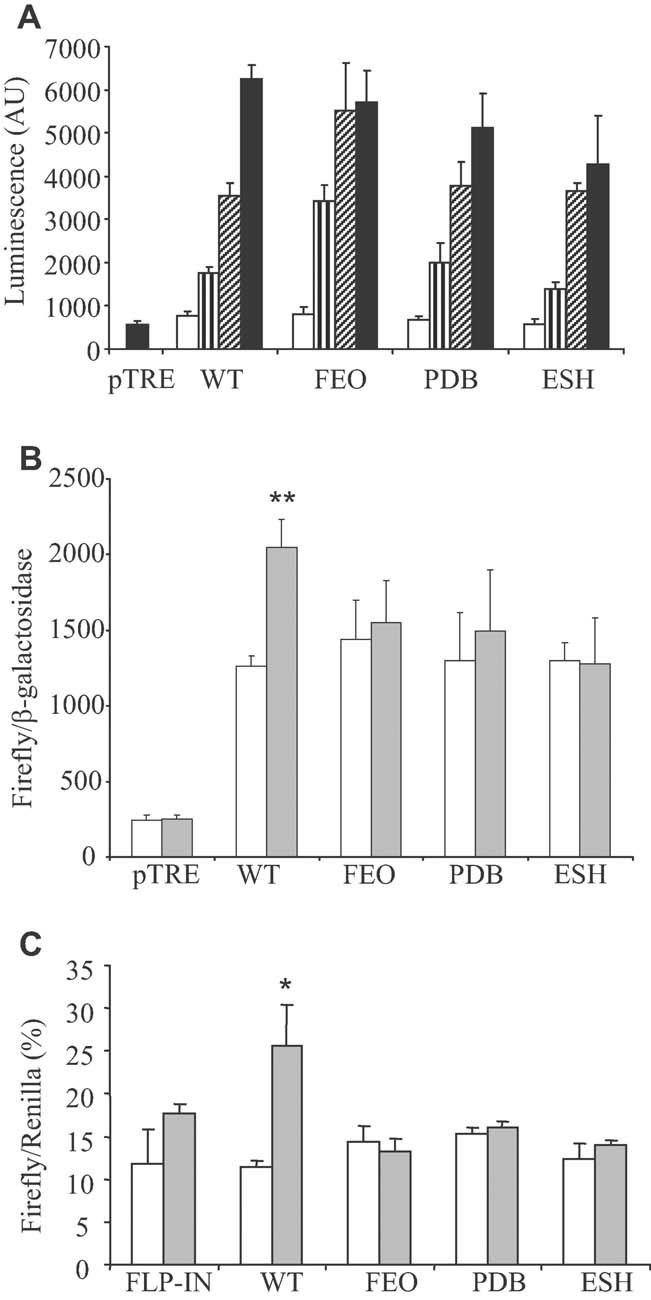
Overexpression of wild-type or mutant RANK proteins in Tet-Off 293 cells constitutively activates NF-κB, but expression of low levels of any of the *RANK* genes does not. In both cases, only cells expressing WT-RANK show NF-κB activation in response to RANKL. (*A*) NF-κB activation was assessed by luciferase reporter assay 48 hours after transfection of Tet-Off-293 cells with 1 ng (*white*), 5 ng (*vertical lines*), 10 ng (*diagonal lines*), or 30 ng (*black*) of RANK constructs together with 150 ng NF-κB–luciferase reporter. Results (mean ± SD, at least four replicates per treatment per experiment) are expressed as relative luminescent activity (arbitrary units). The data are representative of three independent experiments. (*B*) NF-κB activation was assessed by luciferase reporter assay 48 hours after transfection of 293 cells with 5 ng of pTRE-RANK constructs together with 150 ng of NF-κB–luciferase reporter and 150 ng of placZ in the absence (*white bars*) or presence (*gray bars*) of 100 ng/mL of rhRANKL for the final 24 hours of culture. Results (mean ± SD, at least four replicates per treatment per experiment) are expressed as luciferase/β-galactosidase activity. **Significantly different from unstimulated cells, *p* < .001 (one-way ANOVA). The data are representative of three independent experiments. (*C*) NF-κB activation was assessed by luciferase reporter assay in Flp-In, WT-Flp, FEO-Flp, PDB-Flp, and ESH-Flp cells 48 hours after transfection with 150 ng of NF-κB–luciferase reporter and 150 ng of pTK-renilla luciferase in the absence (*white bars*) or presence (*gray bars*) of 100 ng/mL of rhRANKL for the final 24 hours of culture. Results (mean ± SD, at least four replicates per treatment per experiment) are expressed as percentage Firefly/*Renilla* luciferase activity. *Significantly different from unstimulated cells, *p* < .05 (one-way ANOVA). The data are representative of three independent experiments.

Using a luciferase reporter assay to measure NF-κB activation, overexpression of WT-RANK-FLAG, FEO-RANK-FLAG, PDB-RANK-FLAG, and ESH-RANK-FLAG in TetOff293 cells caused constitutive activation of NF-κB. This effect depended on the amount (1 to 30 ng) of *RANK* plasmid transfected into the cells (Fig. 3*A*). As reported previously,^10^ more constitutive activation of NF-κB was detected in cells overexpressing FEO-RANK than WT-RANK, but NF-κB activation in cells overexpressing PDB-RANK or ESH-RANK was not different from that seen in WT-RANK-transfected cells. To study the effect of the *RANK* mutations on RANKL-induced signaling, TetOff293 cells were transfected with just 5 ng of WT-RANK-FLAG, FEO-RANK-FLAG, PDB-RANK-FLAG, or pESH-RANK-FLAG together with 150 ng of pNF-κB-luc and 150 ng of placZ plasmid. This amount of RANK plasmid was selected because it produced a relatively low but significant (*p* < .001, three independent experiments) level of constitutive activation of NF-κB in the experiments described earlier. After 24 hours, the cells were treated with 100 ng/mL of rhRANKL or control medium for a further 24 hours. Only the cells transfected with WT-RANK showed a significant increase (*p* < .001, three independent experiments) in luciferase activity in response to RANKL treatment, demonstrating RANKL-dependent activation of NF-κB via WT-RANK but not via mutant RANK proteins (Fig. 3*B*).

### Flp-WT cells show RANKL-dependent activation of NF-κB

To further distinguish between activation of NF-κB owing to overexpression of RANK proteins and possible activation of NF-κB under more physiologic conditions of lower expression of RANK, Flp-In-293 cells and Flp-WT, Flp-FEO, Flp-PDB, and Flp-ESH cells were cultured in 48-well plates and transfected with 150 ng of pNF-κB-luc and 150 ng of pTKrenilla plasmids. After 24 hours, the cells were cultured with or without 100 ng/mL of rhRANKL for a further 24 hours. NF-κB activation was assessed using a luciferase assay. In the absence of exogenous RANKL, no constitutive activation of NF-κB was detected in any of the RANK-expressing cell lines compared with the untransfected Flp-In-293 cell line (Fig. 3*C*). After RANKL treatment, NF-κB activation in Flp-FEO, Flp-PDB, and Flp-ESH cells was not increased significantly, whereas a significant increase was seen in Flp-WT cells in response to RANKL (*p* < .05, three independent experiments). The same pattern of NF-κB activation also was observed in experiments in which NF-κB signaling was measured 30 and 60 minutes after stimulation with RANKL by using an activated p65 TRANSAM assay (Active Motif, Rixensart, Belgium) (data not shown).

### The subcellular localization of FEO-RANK-FLAG, PDB-RANK-FLAG, and ESH-RANK-FLAG is distinct from that of WT-RANK-FLAG

**Fig. 4:**
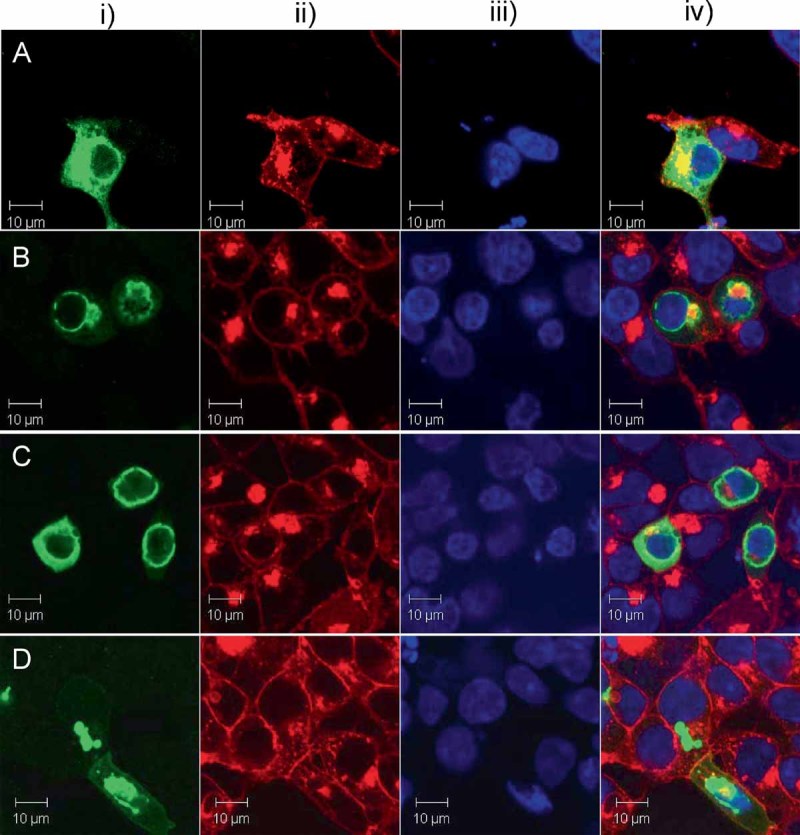
WT-RANK is present in the ER, Golgi, and plasma membrane, but RANK mutants appear to accumulate intracellularly and are not detected predominantly in the Golgi or at the plasma membrane. Thus 293 cells transfected with (*A*) WT-RANK-FLAG, (*B*) FEO-RANK-FLAG, (*C*) PDB-RANK-FLAG, and (*D*) ESH-RANK-FLAG were immunostained for FLAG (*green*, *i*) and stained for Golgi apparatus (wheat germ agglutinin-633; *red*, *iii*), and DNA (Sytox green: *blue*, *iii*) and analyzed by laser scanning confocal microscopy. *xy* sections of representative cells are presented. Panel *iv* in each case represents the merged images. Scale bar = 10 µm.

To study the subcellular localization of mutant RANK, 293 cells were transiently transfected with WT-, FEO-, PDB-, and ESH-RANK-FLAG, immunostained for FLAG, and analyzed by confocal microscopy 24 hours after transfection. WT-RANK-FLAG was detected at the cell surface and in the Golgi apparatus (Fig. 4*A*) because FLAG colocalized with WGA-633, a Golgi marker. FEO-RANK-FLAG and PDB-RANK-FLAG were not detected at the cell surface and appeared in a tight ring around the nucleus and in membrane structures close to the nucleus that did not stain with WGA-633 (Fig. 4*B*, *C*). ESH-RANK-FLAG was detected in vesicular structures throughout the cytosol (Fig. 4*D*) and was not associated with the Golgi.

**Fig. 5:**
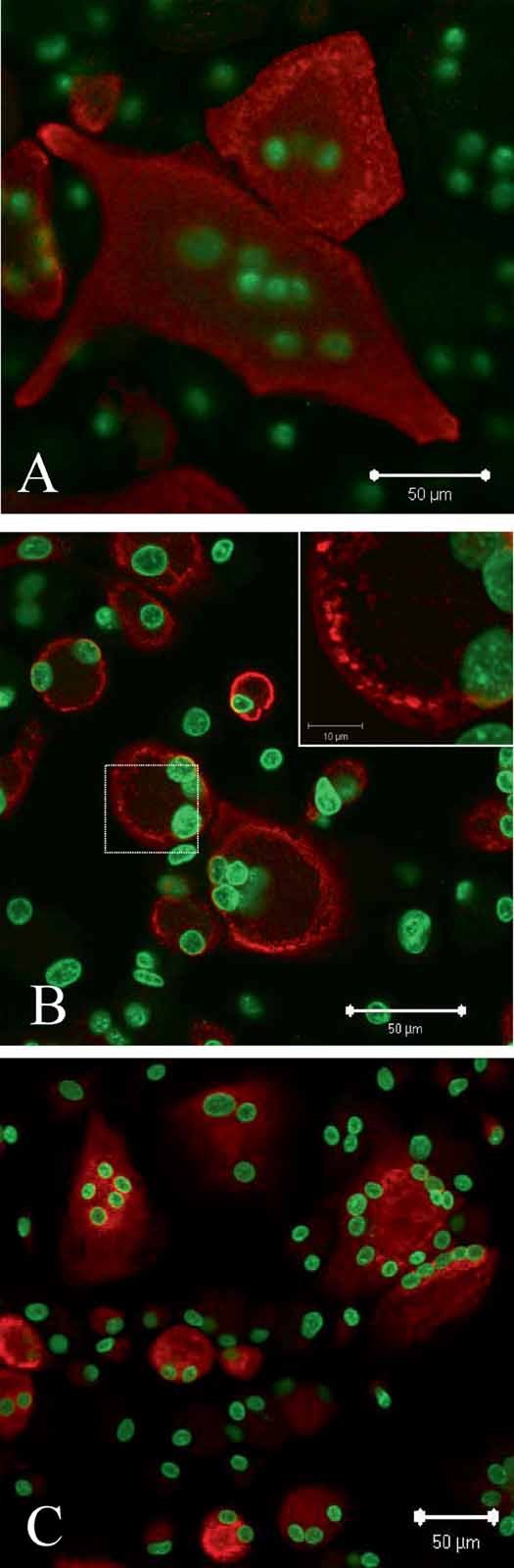
Mutant RANK accumulates intracellularly in human osteoclasts. Human osteoclasts transduced with adenoviral (*A*) WT-RANK-FLAG, (*B*) FEO-RANK-FLAG (including inset magnification of highlighted region), and (*C*) PDB-RANK-FLAG were immunostained for FLAG (*red*) and stained for DNA (Sytox green: *green*) and analyzed by laser scanning confocal microscopy. Images presented are *xy* sections (scale bar shown on each image) of representative cells.

When human osteoclast-like cells were transduced with adenoviral-RANK-FLAG, immunostained for FLAG, and examined by confocal microscopy, WT-RANK-FLAG was detected throughout the cytosol and at the plasma membrane (Fig. 5*A*). By contrast, FEO-RANK-FLAG was detected in a region between the nuclei and the plasma membrane (Fig. 5*B*) and appeared to be associated with small ringlike structures that formed a larger structure surrounding the nuclei (Fig. 5*B*, *inset*), reminiscent of the circular structures that FEO-RANK-FLAG localized to in 293 cells. The pattern of localization of PDB-RANK-FLAG (Fig. 5*C*) was not as distinct as that of FEO-RANK-FLAG and appeared intermediate between that of WT-RANK-FLAG and FEO-RANK-FLAG.

### Localization of WTRANK and mutant RANK proteins by immunoEM

**Fig. 6:**
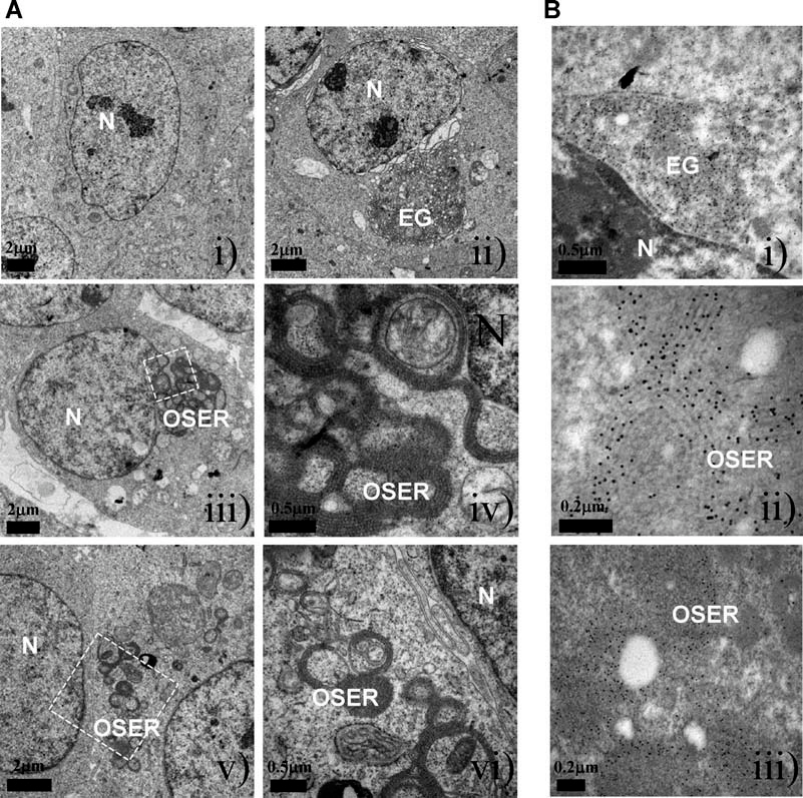
Overexpression of FEO-RANK-FLAG or PDB-RANK-FLAG in 293 cells results in OSER formation. (*A*) TEM analysis at low (*i–iii*, *v*) or high (*iv*, *vi*) magnification of 293 cells that had been (*i*) mock transfected show normal cellular ultrastructure, (*ii*) WT-RANK-FLAG transfected cells show extended Golgi (EG); or (*iii*, *iv*) FEO-RANK-FLAG or (*v*, *vi*) PDB-RANK-FLAG transfected cells show organized smooth endoplasmic reticulum (OSER). Regions highlighted by boxes in *iii* and *v* are shown at higher magnification in *iv* and *vi*. (*B*) ImmunoEM analysis of 293 cells that had been transfected with (*i*) WT-RANK-FLAG show 10-nm gold particles localized to the RER and extended Golgi (EG) or with (*ii*) FEO-RANK-FLAG or (*iii*) PDB-RANK-FLAG show particles localized to OSER. Scale bars are shown on each image. N = nucleus.

To investigate the exact subcellular localization of RANK at the ultrastructural level, HEK 293 cells were transiently transfected with *RANK* expression plasmids. After 24 hours, the cells fixed and processed for conventional TEM or were immunostained using rabbit anti-FLAG primary antibody and 10 nm of gold-labeled secondary antibody and analyzed by TEM. The pattern of staining was very similar to that observed by fluorescence immunostaining. Extended Golgi apparatus could be observed in approximately 20% of osmium-fixed WT-transfected cells analyzed by routine TEM (Fig. 6*A*, *ii*), whereas this was not detected in any empty vector–transfected cells (Fig. 6*A*, *i*). In agreement, gold particles were observed in a tight ring around the nucleus in the rough endoplasmic reticulum and in the Golgi apparatus (Fig. 6*B*, *i*) of WT-transfected cells. When osmium-fixed FEO- and PDB-RANK-transfected cells were analyzed by routine TEM, numerous concentric circular structures were observed within the cytosol of about 26% of the cells examined, continuous with the endoplasmic reticulum and similar to organized smooth endoplasmic reticulum (OSER^16^; Fig. 6*A*, *iii*–*vi*). When this was compared with the FLAG-stained cells, gold particles could be detected only on these membrane structures (Fig. 6*B*, *ii* and *iii*). No OSER was observed in WT-RANK-transfected or cells transfected with empty vector (Fig. 6*A*, *i* and *ii*).

Human osteoclast-like cells grown on nitrocellulose filters and transduced with adeno-WT-RANK-FLAG or adeno-FEO-RANK-FLAG were examined by TEM. In FEO-RANK-FLAG-transduced cells, extensive OSER was observed, consistent with the circular structures observed by confocal microscopy (Fig. 7*B*). These structures were not observed in WT-RANK-FLAG-transduced osteoclasts (Fig. 7*A*) or in untransduced osteoclasts (not shown).

### The signal peptide is not cleaved from FEORANK, PDBRANK, or ESHRANK proteins

**Fig. 8:**
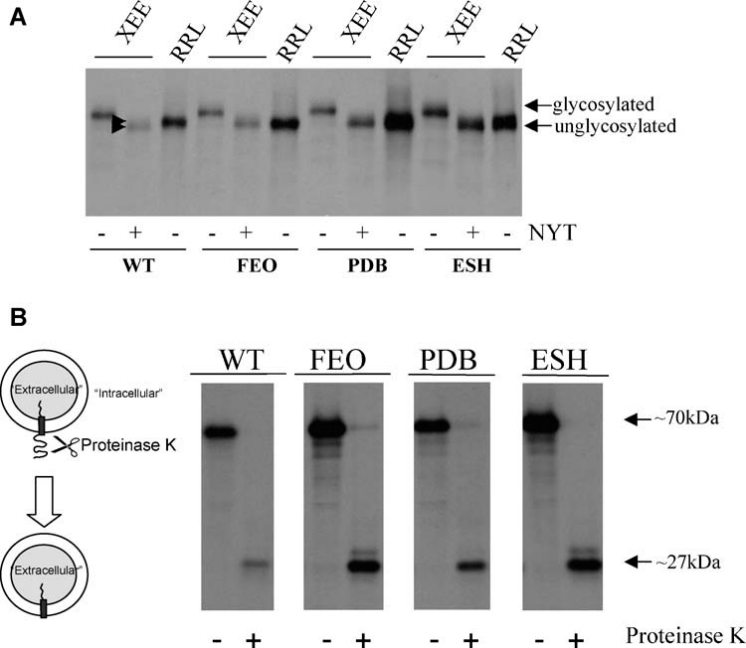
The signal peptide is cleaved from WT-RANK-FLAG but not from FEO-RANK-FLAG, PDB-RANK-FLAG, or ESH-RANK-FLAG. (*A*) All proteins can be glycosylated, as demonstrated when [^35^S]methionine-labeled XEE translation products ± the glysosylation inhibitor NYT were separated on an 8% polyacrylamide gel. Signal peptide cleavage (*two arrowheads*) in WT-RANK-FLAG but not in the mutant proteins can be observed by comparing the size of [^35^S]methionine-labeled XEE translation products in the presence of NYT with RRL translation products. (*B*) Membrane insertion and orientation of the proteins were determined following proteinase K digestion of [^35^S]methionine-labeled XEE translation products from *RANK* RNAs that were separated on a 12.5% polyacrylamide gel. The 27-kDa product that remained in all cases following proteinase K treatment shows that the extracellular domain was protected from digestion (illustrated by the schematic representation), and therefore, all the proteins are inserted in the correct orientation in the membrane.

To investigate signal peptide cleavage, we compared the molecular mass of proteins synthesised in the *Xenopus* egg extract (XEE) system with the mass of proteins synthesized in the rabbit reticulocyte system. The XEE system is a homologous coupled translation/translocation system that allows membrane insertion (including signal peptide cleavage) and posttranslational modifications (including glycosylation).^15^ By contrast, the rabbit reticulocyte lysate (RRL) does not contain any membranes, and therefore translated products are not posttranslationally modified.^17^ By using the glycolysation inhibitor NYT in the XEE system, we were able to directly compare the mass of proteins translated in the two systems in the absence of posttranslational glycosylation. Whereas WT-RANK-FLAG was of lower mass when synthesized in the XEE system compared with the RRL system, FEO-, PDB- and ESH-RANK-FLAG proteins were of the same mass in both translation systems (Fig. 8*A*), indicating that the signal peptide is not cleaved from the mutant proteins. This is so despite the fact that the mutant proteins still can be glycosylated (Fig. 8*A*), as demonstrated by a shift in protein mass in the presence/absence of NYT in the XEE system. These data, together with the evidence for lack of ligand-dependent signaling downstream of FEO-, PDB-, and ESH-RANK, strongly suggest that the signal peptide is not cleaved from the mutant proteins and that this prevents plasma membrane localization of the mutant proteins. Finally, despite the lack of signal peptide cleavage, the mutant proteins still could be inserted into the membrane and in the correct orientation in the XEE system, as demonstrated by a protease protection assay showing the shift in molecular mass from approximately 70 to 27 kDa. This demonstrates that the extracellular domain of RANK was protected from proteinase K digestion in all proteins (Fig. 8*B*).

## Discussion

RANK is present as a transmembrane receptor at the plasma membrane of osteoclasts and their precursors. Oligomerization, via a cytoplasmic oligomerization motif, is an absolute requirement for osteoclast formation but can occur in the absence of RANKL.^18^ Signaling from the RANK receptor, via NF-κB, NFATc1, and AP-1, depends on several cytoplasmic domains of the RANK protein^19^, ^20^ that are important for its interaction with downstream mediators, including TRAF6.

FEO, ePDB, and ESH are closely related skeletal disorders^12^, ^21^, ^22^ that are sometimes characterized by localized osteolytic lesions as a result of hyperactive osteoclasts on a background of increased generalized bone turnover. The disorders are caused by insertion mutations in the signal peptide of RANK that cause amino acid duplications of 6 (FEO, from residue 21), 9 (ePDB, from residue 18), and 5 (ESH, from residue 21), respectively.^10^, ^11^ The exact mechanism by which the *RANK* mutations affect downstream signaling pathways remains unclear. To investigate the outcome of carriage of these three *RANK* mutations on the processing and signaling via RANK, we overexpressed the mutant proteins in cell lines and primary human osteoclast-like cells.

In the initial study by Hughes and colleagues, it was suggested, following overexpression studies in EBNA293 cells, that FEO-RANK and PDB-RANK caused constitutive activation of NF-κB, thereby leading to the characteristic overactive osteoclast phenotype.^10^ Although we also found that overexpression of FEO-RANK and PDB-RANK, as well as ESH-RANK, results in constitutive activation of NF-κB in 293 cells, we found that NF-κB was not constitutively activated in a more physiologic model (293 cells stably transfected with a single copy of the *RANK* genes), suggesting that the constitutive activation of NF-κB seen in overexpression models could be as a result of ER overload. The latter effect is known to cause NF-κB activation when any transmembrane protein is overexpressed.^23^ Ligand-dependent activation of NF-κB occurred only in cells expressing WT-RANK-FLAG (either overexpression or physiologic expression), providing further evidence of lack of plasma membrane localization of the mutant RANK proteins.

Alternatively, constitutive activation of NF-κB in the presence of overexpressed RANK proteins could occur as a result of a RANK-dependent mechanism. Since the transmembrane domain of RANK is predicted to be at positions 201–222, it is expected that the N-terminal extracellular domain (approximately 400 amino acids) should be protected from proteinase K treatment in the protease protection assay that we employed (because it will be within the lumen of the membrane vesicles containing the newly translated/translocated protein). This was confirmed when the [^35^S]methionine-labeled protein was reduced from approximately 600 amino acids to approximately 200 amino acids following proteinase K digestion. Since WT-RANK-FLAG and the three mutant RANK proteins were inserted in the expected orientation within the membrane (ie, C-terminus in the cytosol), this leaves the possibility that signaling proteins (such as TRAF6) still may interact with the C-terminal domains of RANK, even if the protein is trapped in the ER. However, although we have observed that the wild-type and mutant RANK proteins can interact with TRAF6 as measured by immunoprecipitation, when cells overexpressing the RANK constructs were cotransfected with a construct expressing a dominant-negative form of TRAF6, there was no effect on the constitutive levels of NF-κB activation, but ligand-dependent activation of NF-κB was at least partially prevented (data not shown).

As an integral plasma membrane protein, RANK is predicted to be translated and translocated into the rough endoplasmic reticulum (RER), where the signal peptide is removed by signal peptidase [predicted by SignalP 3.0 to occur between amino acid residues 27 (Ala) and 28 (Leu)] and where glycosylation can occur on the two predicted N-terminal glycosylation motifs (Asn-X-Ser/Thr). As expected, WT-RANK-FLAG was detected both within the Golgi apparatus and at the plasma membrane of 293 cells and transduced human osteoclasts by immunostaining. By contrast, RANK proteins carrying the FEO, PDB, and ESH mutations were not detected in the Golgi apparatus or at the plasma membrane, but in transfected 293 cells and transduced osteoclasts, they accumulated within multilamellar extensions of the ER reminiscent of OSER, a feature associated with the overexpression of ER-resident transmembrane proteins.^16^, ^24^ This is relevant because, as we demonstrate here, the insertion mutations in *RANK* prevent cleavage of the signal peptide, making the protein effectively an ER-resident protein. This is in agreement with Hughes and colleagues, who predicted that the signal peptides within FEO-RANK and PDB-RANK were not cleaved based on SignalP 3.0 data and differences in molecular mass compared with WT-RANK.^10^ Accumulation of both FEO-RANK and PDB-RANK proteins caused OSER formation—in FEO-RANK-transfected cells, the OSER structures seemed to be continuous with the RER and predominantly perinuclear, whereas PDB caused OSER formation throughout the cytoplasm. All OSER formed was of a lamellar type when compared with previous studies of other overexpressed ER-resident proteins.^16^ Formation of OSER was clearly associated with overexpression of mutant RANK protein because no OSER was detected in cells transfected with WTRANK or in osteoclasts transduced with empty vector. Furthermore, FLAG staining was almost exclusively localized to the OSER structures in FEO-RANK- and PDB-RANK- transfected 293 cells analyzed by immunoEM. In agreement with OSER formation being a feature of protein overexpression, it was not detected in the stable cell lines expressing just a single copy of WT-RANK, FEO-RANK, PDB-RANK, or ESH-RANK (data not shown), and OSER has not been described in human bone biopsies in FEO, PDB, or ESH patients. Furthermore, OSER does not resemble the cytoplasmic inclusions that have been observed in the osteoclasts of some FEO patients, the exact origin of which is still a matter of debate between laboratories that suggest they are evidence of previous exposure to paramyoxiviridae and those which have been unable to detect viral transcripts within any pagetic patient samples tested.^25^ Thus, although formation of OSER appears to be an artifact of overexpression of the RANK mutants, this phenomenon clearly demonstrates that the mutations cause localization of RANK in the ER and prevent its targeting to the plasma membrane.

Taken together, these data provide convincing evidence that the signal peptide mutations in the *RANK* gene result in functional *inactivation* of the RANK signaling pathway because of lack of translocation of the receptor to the plasma membrane. These observations are supported by recent phenotypic characterization of a transgenic knock-in mouse model engineered to express *Rank* containing the PDB mutation.^26^ While homozygous mice had an osteopetrotic phenotype consistent with complete absence of functional RANK protein, heterozygotes showed severe osteolytic lesions and represent a phenocopy of ePDB patients. While our studies show that constitutive activation of NF-κB in cells is only a feature of cells that overexpress either wild-type or mutant RANK proteins, we do not exclude the possibility that in patient cells there may be increased RANK-dependent signaling as a result of interaction between the wild-type protein and the mutant protein that may affect the regulation of signaling downstream from the receptor. Further studies on RANK signaling in heterozygous models of wild-type and mutant RANK, including in vivo models, are clearly required to fully understand how signal peptide mutations in *RANK* cause osteoclast overactivity.

## Disclosures

All the authors state that they have no conflicts of interest.
